# The impact of hypertension on clinical outcomes in moyamoya disease: a multicenter, propensity score-matched analysis

**DOI:** 10.1007/s00701-024-06254-0

**Published:** 2024-09-13

**Authors:** Basel Musmar, Joanna M. Roy, Hammam Abdalrazeq, Elias Atallah, Kareem El Naamani, Ching-Jen Chen, Roland Jabre, Hassan Saad, Jonathan A. Grossberg, Adam A. Dmytriw, Aman B. Patel, Mirhojjat Khorasanizadeh, Christopher S Ogilvy, Ajith J. Thomas, Andre Monteiro, Adnan Siddiqui, Gustavo M. Cortez, Ricardo A. Hanel, Guilherme Porto, Alejandro M. Spiotta, Anthony J. Piscopo, David M. Hasan, Mohammad Ghorbani, Joshua Weinberg, Shahid M. Nimjee, Kimon Bekelis, Mohamed M. Salem, Jan-Karl Burkhardt, Akli Zetchi, Charles Matouk, Brian M. Howard, Rosalind Lai, Rose Du, Rawad Abbas, Georgios S Sioutas, Abdelaziz Amllay, Alfredo Munoz, Nabeel A. Herial, Stavropoula I. Tjoumakaris, Michael Reid Gooch, Robert H. Rosenwasser, Pascal Jabbour

**Affiliations:** 1 Department of Neurological Surgery, Thomas Jefferson University Hospital, 901 Walnut street 3rd Floor, Philadelphia, Pennsylvania 19107 USA; 2 Department of Neurosurgery, University of Arizona college of medicine, Tucson, Arizona USA; 3 Department of Neurosurgery, The University of Texas Health Science Center, Houston, TX USA; 4 Department of Neurosurgery, Emory University, Atlanta, Georgia USA; 5 Department of Medical Imaging, University of Toronto Faculty of Medicine, Toronto, Ontario Canada; 6 Neuroendovascular Program, Massachusetts General Hospital & Brigham and Women’s Hospital, Harvard Medical School, Boston, MA USA; 7 Department of Neurosurgery, Beth Israel Deaconess Medical Center and Harvard Medical School, Boston, MA USA; 8 Department of Neurological Surgery, Cooper University Health Care, Cooper Medical School of Rowan University, Camden, NJ USA; 9 Department of Neurosurgery, University of New York at Buffalo, Buffalo, NY USA; 10 Lyerly Neurosurgery, Baptist Health System, Jacksonville, FL USA; 11 Department of Neurosurgery and Neuroendovascular Surgery, Medical University of South Carolina, Charleston, SC USA; 12 Department of Neurosurgery, University of Iowa Hospital and Clinics, Iowa City, IA USA; 13 Department of Neurosurgery, Duke University, Durham, NC USA; 14 Department of Neurosurgery, Firoozgar Hospital, Tehran, Iran; 15 Department of Neurosurgery, The Ohio State University Wexner Medical Center, Columbus, OH USA; 16 Good Samaritan Hospital Medical Center, Babylon, NY USA; 17 Department of Neurosurgery, Hospital of the University of Pennsylvania, Penn Medicine, Philadelphia, PA USA; 18 Department of Neurosurgery, Yale University, New Haven, CT USA; 19 Department of Neurosurgery and of Radiology and Biomedical Imaging, Yale University, New Haven, CT USA

**Keywords:** Moyamoya, HTN, Stroke, Multicenter

## Abstract

**Background:**

Moyamoya disease (MMD) is a rare cerebrovascular disorder characterized by progressive steno-occlusive changes in the internal carotid arteries, leading to an abnormal vascular network. Hypertension is prevalent among MMD patients, raising concerns about its impact on disease outcomes. This study aims to compare the clinical characteristics and outcomes of MMD patients with and without hypertension.

**Methods:**

We conducted a multicenter, retrospective study involving 598 MMD patients who underwent surgical revascularization across 13 academic institutions in North America. Patients were categorized into hypertensive (*n=*292) and non-hypertensive (*n=*306) cohorts. Propensity score matching (PSM) was performed to adjust for baseline differences.

**Results:**

The mean age was higher in the hypertension group (46 years vs. 36.8 years, *p <* 0.001). Hypertensive patients had higher rates of diabetes mellitus (45.2% vs. 10.7%, *p <* 0.001) and smoking (48.8% vs. 27.1%, *p <* 0.001). Symptomatic stroke rates were higher in the hypertension group (16% vs. 7.1%; OR: 2.48; 95% CI: 1.39-4.40, *p =* 0.002) before matching. After PSM, there were no significant differences in symptomatic stroke rates (11.1% vs. 7.7%; OR: 1.5; CI: 0.64-3.47, *p =* 0.34), perioperative strokes (6.2% vs. 2.1%; OR 3.13; 95% CI: 0.83-11.82, *p =* 0.09), or good functional outcomes at discharge (93% vs. 92.3%; OR 1.1; 95% CI: 0.45-2.69, *p =* 0.82).

**Conclusion:**

No significant differences in symptomatic stroke rates, perioperative strokes, or functional outcomes were observed between hypertensive and non-hypertensive Moyamoya patients. Appropriate management can lead to similar outcomes in both groups. Further prospective studies are required to validate these findings.

**Supplementary Information:**

The online version contains supplementary material available at 10.1007/s00701-024-06254-0.

## Introduction

Moyamoya disease (MMD) is a rare cerebrovascular disorder characterized by progressive steno-occlusive changes in the terminal portion of the internal carotid arteries and their main branches [23]. This pathological process leads to the formation of an abnormal vascular network at the base of the brain, which appears as a "puff of smoke" on angiography [23]. Although the etiology of MMD remains unclear, the disease manifests in a bimodal distribution, primarily affecting children aged 5-14 and adults aged 45-54 [12].

Clinical presentations of MMD are diverse, including strokes, transient ischemic attacks (TIA), seizures, aphasia, headaches, cognitive impairments in children, dysarthria, and hemiparesis [20]. Diagnostic modalities for MMD include CT angiography (CTA), magnetic resonance imaging (MRI), and MR angiography (MRA), with conventional angiography remaining the gold standard for both diagnosis and surgical planning [4]. Management of MMD primarily involves revascularization procedures, which aim to prevent stroke by enhancing cerebral blood flow in affected areas [1, 6, 15, 17].

It has been observed that a subset of MMD patients presents with renovascular hypertension, a condition associated with renal artery lesions [13, 19, 21]. The prevalence of renovascular hypertension in MMD patients is estimated to be around 2% [12, 27]. However, clinical observations suggest a higher prevalence of hypertension among MMD patients, raising concerns about its impact on disease outcomes [16].

Therefore, we aim to compare the clinical characteristics and outcomes of Moyamoya disease patients with and without hypertension using a multicenter, institutional, propensity score-matched analysis.

## Methods

We conducted a multicenter, retrospective study in accordance with the Strengthening the Reporting of Observational Studies in Epidemiology (STROBE) guidelines [5]. Institutional review board approval was obtained at all centers. No identifiable patient information was presented in the study and, thus, informed consent was not required.

### Patient population

This study involved Moyamoya-affected hemispheres treated with surgical revascularization across 13 academic institutions predominantly in North America. Inclusion criteria were standardized across centers and included all patients with Moyamoya disease who underwent surgical revascularization treatment. Data were collected and analyzed on a per-hemisphere basis, categorizing hemispheres into hypertensive (above 139/89) and non-hypertensive (120/80 to 139/89) cohorts based on patient medical history. Hypertension was defined as a documented history of hypertension (systolic blood pressure >139 mmHg or diastolic blood pressure >89 mmHg) or the use of antihypertensive medications at the time of admission [26]. Patients with secondary causes of systemic hypertension, such as renal artery stenosis or endocrine disorders, were excluded from the analysis to focus on primary hypertension.

Data collected included patient demographics (age, gender, race, hypertension, diabetes mellitus, smoking status, sickle cell disease), presenting symptoms (TIA, stroke, subarachnoid hemorrhage (SAH), intraparenchymal hemorrhage, intraventricular hemorrhage (IVH), incidental finding), disease characteristics (laterality, Suzuki grade), procedural details (DR vs IR), complications (major, minor, hemorrhagic, ischemic, periprocedural), follow-up (length of follow-up), and angiographic and functional outcomes (modified Rankin Score (mRS) and National Institute of Health Stroke Scale (NIHSS)).

### Study endpoints

Study outcomes included major (ischemic or hemorrhagic with >4 change in NIHSS score) and minor (ischemic or hemorrhagic with <4 change in NIHSS score) symptomatic strokes (confirmed by imaging), good functional outcome (mRS 0-2) at discharge, NIHSS at discharge, length of hospital stay (days), perioperative strokes (including minor and major strokes confirmed by imaging), and follow-up strokes, categorized into ischemic and hemorrhagic after discharge. A stroke was defined by a new hypodensity on CT or a diffusion-weighted imaging hit on MRI not present on admission. A TIA was defined by a transient acute neurological deficit lasting less than 24 hours without radiographic evidence of stroke.

### Statistical analysis

All statistical analyses were conducted using Stata (V.17.0; StataCorp). Baseline characteristics of hypertensive and non-hypertensive cohorts were compared using Pearson’s chi-squared or Fisher’s exact tests for categorical variables, and Student’s t-test or Mann-Whitney U tests for continuous variables, as appropriate. Given the significant baseline differences between hypertensive and non-hypertensive patients—such as age, diabetes mellitus, and smoking status—we used propensity score matching (PSM) to control for these confounders [2]. PSM was performed in a 1:1 ratio without replacement, using a caliper of 0.2 standard deviations of the logit of the propensity score. Propensity scores were derived using a logistic regression model that accounted for all baseline characteristics. The PSMATCH2 package for Stata was utilized for the propensity score derivation [14].

Outcome differences between hypertensive and non-hypertensive cohorts, both before and after matching, were assessed using univariable binary logistic and linear regression analyses, as appropriate. Results were reported as odds ratios (ORs) or beta coefficients with corresponding 95% confidence intervals (CIs). Fisher’s exact test was used for comparing outcomes with zero frequencies. Statistical significance was set at *p*<0.05, and all tests were two-tailed. Because the number of missing data points was minimal, no imputation was performed to avoid introducing bias [11]. The analysis was conducted using available data only.

We also used a Cox Proportional Hazard Model to determine the effect of hypertension in both symptomatic stroke and follow-up stroke. The model was adjusted to age, smoking, Suzuki grade, procedure type, diabetes mellitus, underlying disease, surgery side, and incidental MMD.

## Results

### Baseline characteristics

**Fig. 1:**
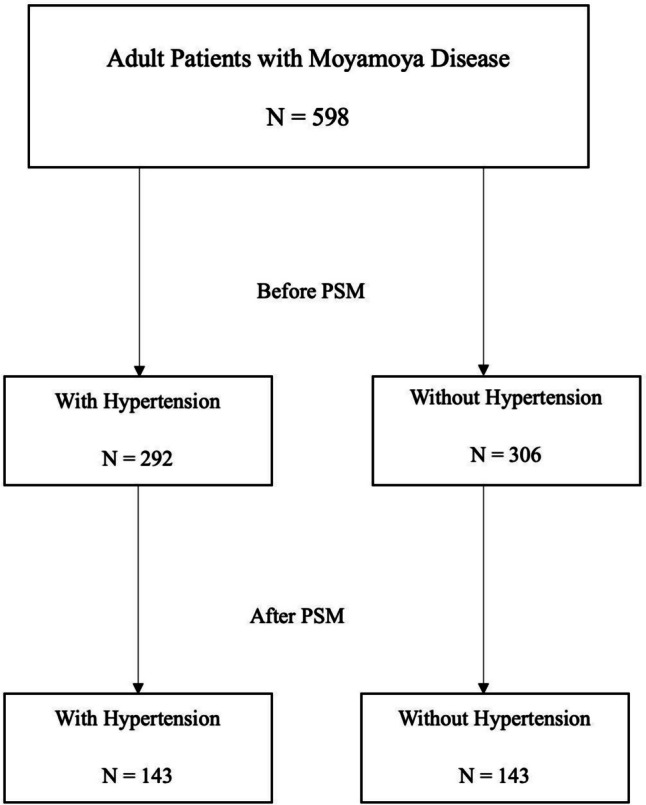
Flowchart shows the inclusion for patients in this study

A total of 598 patients were included, with 292 patients having hypertension and 306 patients without hypertension (Fig. 1). The mean age was significantly higher in the hypertension group (46 years, SD 12.5) compared to the non-hypertension group (36.8 years, SD 15.1) (*p <* 0.001). The gender distribution was similar between the two groups, with 29.7% of males in the hypertension group and 29% in the non-hypertension group (*p =* 0.84).

Comorbid conditions were more prevalent in the hypertension group, with higher rates of diabetes mellitus (45.2% vs. 10.7%, *p <* 0.001) and smoking (48.8% vs. 27.1%, *p <* 0.001). There was no significant difference in the family history of Moyamoya disease (2% vs. 1.3%, *p =* 0.53), but the prevalence of sickle cell disease was lower in the hypertension group (3.4% vs. 7.5%, *p =* 0.03). The rates of indirect revascularization were similar between the two groups (60.2% vs. 57.1%, *p =* 0.45). However, hypertensive moyamoya patients’ group had a higher rate of combined revascularization compared to the non-hypertensive moyamoya patients’ group (13.3% vs. 6.2%, *p =* 0.004) (Table 1).

**Table 1:** Comparison of baseline characteristics between unmatched patients with and without hypertension

	Total = 598	With hypertension = 292	Without hypertension = 306	*P*-value
Age, mean years (SD)	41.3 (14.6)	46.0 (12.5)	36.8 (15.1)	**< 0.001**
Male, n (%)	176/598 (29.4)	87/292 (29.7)	90/306(29.0)	0.84
Race, n (%)				
Caucasian	318/598 (53.1)	148/292 (50.6)	170/306 (55.5)	0.23
African-American	170/598 (28.4)	93/292(31.8)	77/306 (25.1)	0.07
Asian	70/598 (11.7)	33/292 (11.3)	37/306 (12.0)	0.80
Hispanic	27/598 (45)	10/292 (3.4)	17/306 (9.1)	0.24
other	13/598 (2.1)	8/292 (2.7)	5/306 1.6)	0.40
Diabetes mellitus, n (%)	165/598 (27.5)	132/292 (45.2)	33/306 (10.7)	**< 0.001**
Smoker, n (%)	214/598 (35.7)	131/292 (48.8)	83/306 (27.1)	**< 0.001**
Family history of moyamoya, n (%)	10/598 (1.6)	6/292 (2.0)	4/306 (1.3)	0.53
Underlying disease, n (%				
Sickle cell disease	33/598 (5.5)	10/292 (3.4)	23/306 (7.5)	**0.03**
Sickle cell trait	5/598 (0.8)	3/292 (1.3)	2/306 (0.6)	0.67
Neurofibromatosis	4/598 (0.67)	3/292 (1.0)	1/306 (0.3)	0.36
Procedure type, n (%)				
Indirect Revascularization	351/598 (58.7)	176/292 (60.2)	175/306 (57.1)	0.45
Direct Revascularization	305/598 (51.0)	155/292 (53.0)	150/306 (49.0)	0.32
Combined	58/598 (9.7)	39/292 (13.3)	19/306 (6.2)	**0.004**
Suzuki grade, n (%)				
I	24/598 (4.0)	12/289 (4.1)	12/303 (3.9)	1.0
II	71/598 (11.8)	31/292 (10.6)	40/306 (13.0)	0.37
III	181/598 (30.2)	95/292 (32.5)	86/306 (28.1)	0.23
IV	178/598 (29.7)	83/292 (28.4)	95/306 (31.0)	0.48
V	95/598 (15.8)	43/292 (14.7)	52/306 (16.9)	0.50
VI	41/598 (6.8)	24/292 (8.2)	17/306 (5.5)	0.25
Surgery side, n (%)				
Right hemisphere	306/598 (51.1)	148/292 (50.6)	158/306 (51.6)	0.81
Left hemisphere	292/598 (48.8)	144/292 (49.3)	148/306 (48.3)	0.81
Follow-up (months), median months (IQR)	17 (7-54)	16 (7-50)	17 (7-57)	0.64
mRS (0-2) on admission, n (%)	529/590 (89.6)	254/287 (88.5)	275/303 (90.7)	0.41
Incidental, n (%)	163/598 (27.2)	68/292 (23.2)	95/306 (31.0)	**0.03**
Stroke, n%				
Ischemic stroke	339/598 (56.6)	172/292 (58.9)	167/306 (54.5)	0.28
TIA	129/598 (21.5)	58/292 (19.8)	71/306 (23.2)	0.37
Intraventricular hemorrhage	15/598 (2.5)	10/292 (3.4)	5/306 (1.6)	0.19
Intracerebral hemorrhage	38/598 (6.3)	19/292 (6.5)	19/306 (6.2)	1.0
Subarachnoid hemorrhage	35/598 (5.8)	18/292 (6.1)	17/306 (5.5)	0.86

### Outcomes

**Fig. 2:**
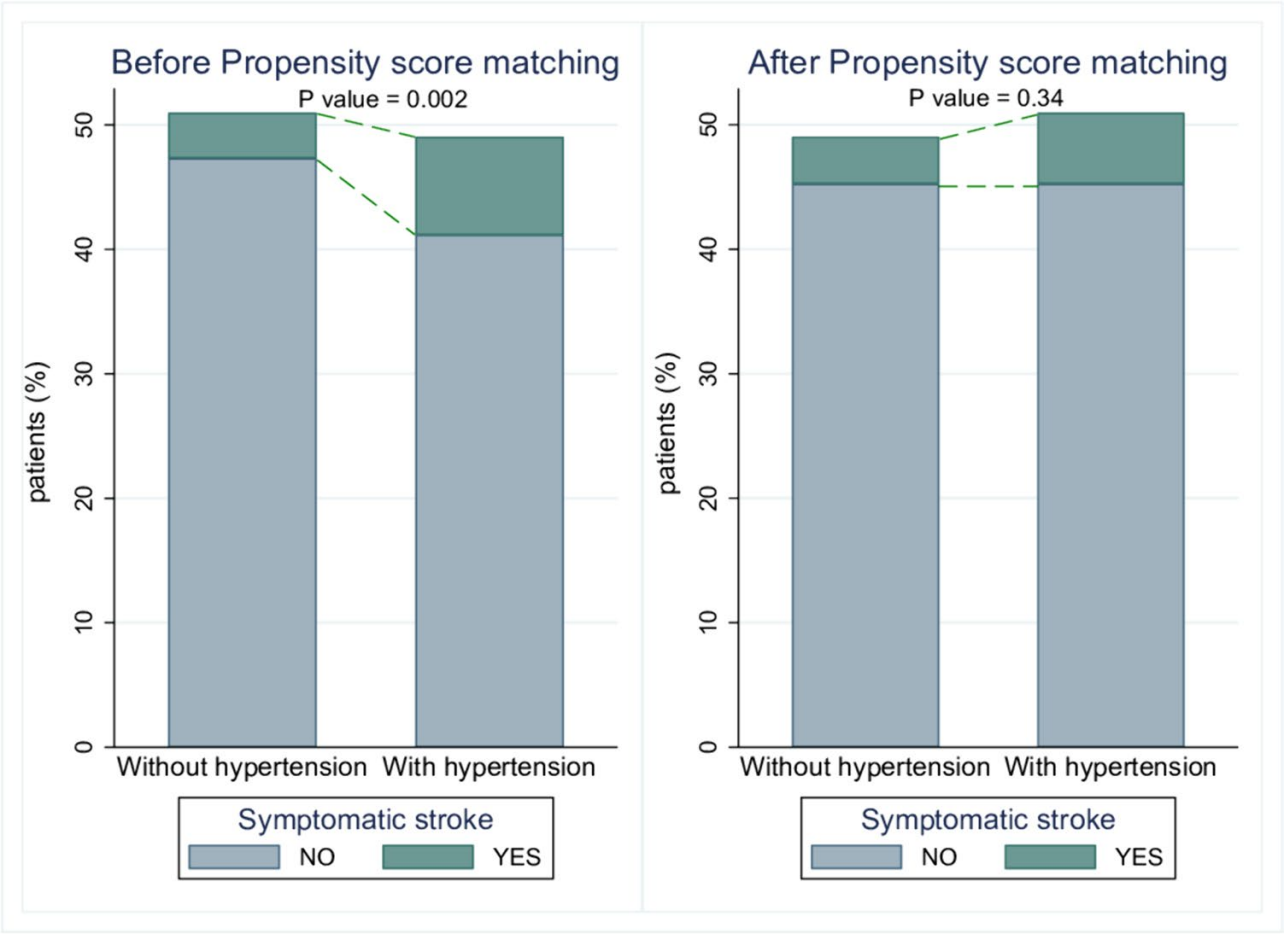
Incidence of symptomatic stroke in Moyamoya disease patients with and without hypertension, before and after PSM

**Fig. 3:**
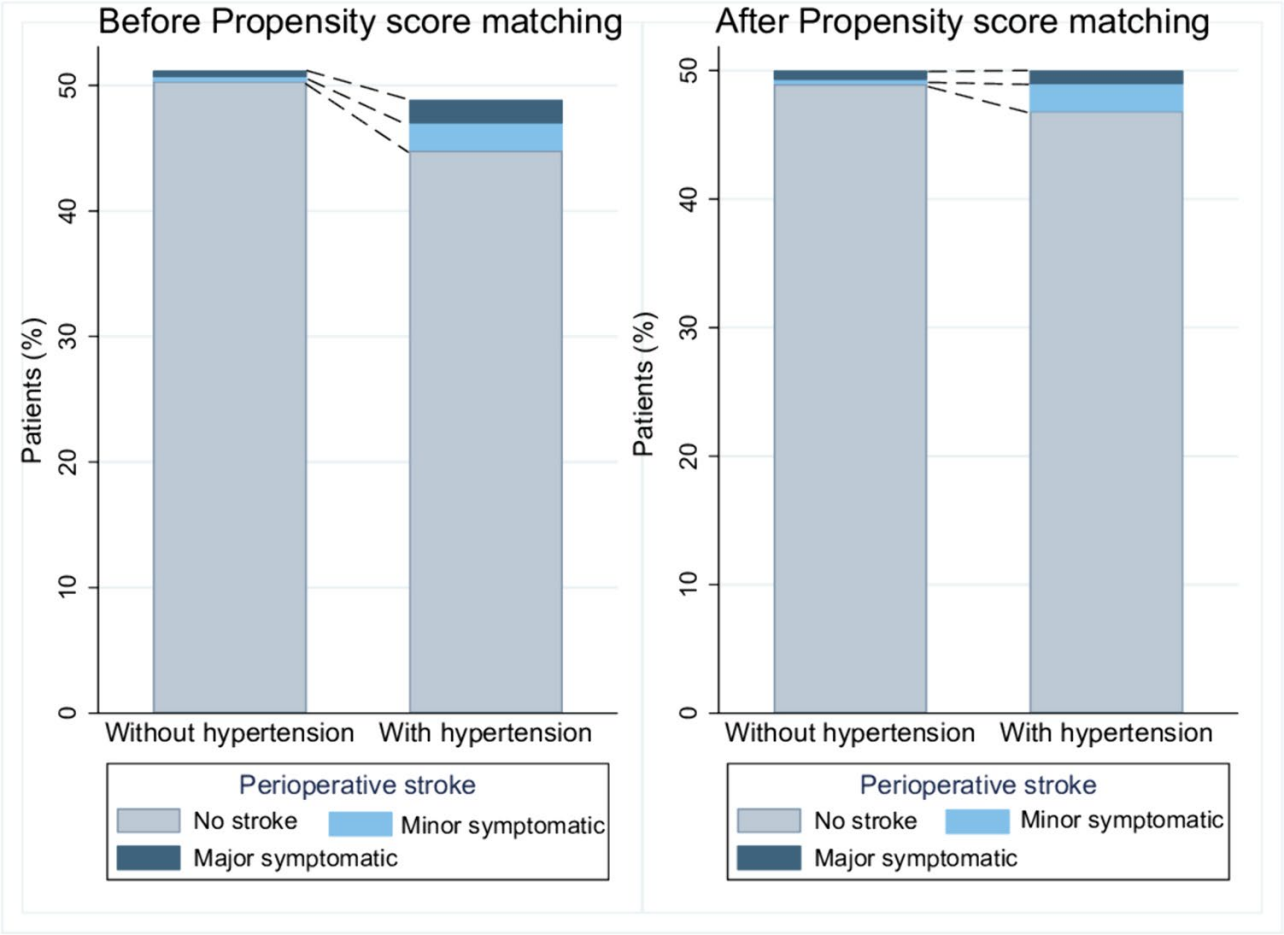
Perioperative stroke outcomes in Moyamoya disease patients with and without hypertension, before and after PSM

The overall symptomatic stroke occurred more frequently in the hypertension group (16% vs. 7.1%; OR: 2.48; 95% CI: 1.39-4.40, *p =* 0.002) (Fig. 2). Symptomatic ischemic strokes were more common in the hypertension group (14.5% vs. 6%; OR: 2.62; CI: 1.41-4.48, *p =* 0.002). Moreover, the hypertension group had a higher rate of perioperative stroke (8.2% vs. 2.2%; OR: 3.82; CI: 1.62-9.02, *p =* 0.002) including minor symptomatic (4.4% vs. 0.6%; OR: 7.08; CI: 1.58-31.66, *p =* 0.01) and major symptomatic (3.7% vs. 0.9%; OR: 3.95; CI: 1.09-14.31, *p =* 0.036) compared to the non-hypertension group (Fig. 3).

Good functional outcome at discharge, measured by the mRS score, was similar between the groups (91% in hypertension vs. 92.4% in non-hypertension; OR 0.83; 95% CI: 0.47-1.50, *p =* 0.55). NIHSS scores at discharge were comparable (median 0 in both groups, *p =* 0.76). Length of hospital stay was slightly longer in the hypertension group but did not reach statistical significance (median 4 days vs. 3 days, *p =* 0.06). Follow-up stroke rates were higher in the hypertension group but were not statistically significant (9.5% vs. 6.8%; OR 1.45; 95% CI: 0.79-2.67, *p =* 0.22) (Table 2).

**Table 2:** Comparison of outcomes between unmatched patients with and without hypertension

	With hypertension = 292	Without hypertension = 306	Effect variable	Value (95% CI)	*P*-value
Symptomatic stroke, n (%)	41/252 (16.0)	19/263 (7.1)	OR	2.48 (1.39 to 4.40)	**0.002**
Symptomatic ischemic stroke, n (%)	37/255 (14.5)	16/263 (6.0)	OR	2.62 (1.41 to 4.48)	**0.002**
Symptomatic hemorrhagic stroke, n (%)	5/252 (1.9)	1/263 (0.3)	OR	5.30 (0.61 to 45.71)	0.12
Intraoperative complication, n (%)	31/292 (10.6)	21/306 (6.8)	OR	1.61 (0.90 to 2.87)	0.10
Perioperative stroke, n (%)	24/292 (8.2)	7/306 (2.2)	OR	3.82 (1.62 to 9.02)	**0.002**
Perioperative minor symptomatic stroke, n (%)	13/292 (4.4)	2/306 (0.6)	OR	7.08 (1.58 to 31.66)	**0.01**
Perioperative major symptomatic stroke, n (%)	11/292 (3.7)	3/306 (0.9)	OR	3.95 (1.09 to 14.31)	**0.036**
Good functional outcome at discharge, n (%)	264/290 (91.0)	281/303 (92.4)	OR	0.83 (0.47 to1.50)	0.55
NIHSS at discharge, median (IQR)	0 (0-1)	0 (0-1)	Beta	-0.005 (-0.04 to 0.03)	0.76
Length of hospital stay, median (IQR)	4 (2-6)	3 (2-5)	Beta	0.02 (-0.00 to 0.06)	0.06
Follow-up stroke, n (%)	26/271 (9.5)	20/294 (6.8)	OR	1.45 (0.79 to 2.67)	0.22
Follow-up ischemic stroke, n (%)	23/292 (7.8)	18/306 (5.8)	OR	1.36 (0.72 to 2.59)	0.33
Follow-up hemorrhagic stroke, n (%)	3/292 (1.0)	2/306 (0.6)	OR	1.57 (0.26 to 9.51)	0.50

### Propensity score matching

PSM resulted in 143 matched pairs (Table 3) (Fig. 1). Although symptomatic stroke occurred more in the hypertensive group, it didn’t reach statistical significance (11.1% vs. 7.7%; OR: 1.5; CI: 0.64-3.47, *p =* 0.34) (Fig. 2). Similarly, perioperative strokes were more common in the hypertension group but were not statistically significant (6.2% vs. 2.1%; OR 3.13; 95% CI: 0.83-11.82, *p =* 0.09) (Fig. 3). Good functional outcome at discharge was similar between the groups (93% in hypertension vs. 92.3% in non-hypertension; OR 1.1; 95% CI: 0.45-2.69, *p =* 0.82). NIHSS scores at discharge were comparable (median 0 in both groups, *p =* 0.86). Length of hospital stay was not significantly different (median 4 days vs. 3 days, *p =* 0.91). Follow-up stroke rates were similar between the groups (6.2% in hypertension vs. 5.5% in non-hypertension; OR 1.13; 95% CI: 0.42-3.02, *p =* 0.8) (Table 4).

**Table 3:** Comparison of baseline characteristics between matched patients with and without hypertension

	Total = 286	With hypertension = 143	Without hypertension = 143	*P*-value
Age, mean years (SD)	42.5 (14.4)	42.5 (12.2)	42.5 (12.7)	0.96
Male, n (%)	73/286 (25.5)	38/143 (26.5)	35/143 (24.4)	0.68
Race, n (%)				
Caucasian	166/286 (58.0)	48/143 (58.7)	82/143 (57.3)	0.90
African-American	82/286 (28.6)	41/143 (28.6)	41/143 (28.6)	1.00
Asian	21/286 (7.3)	10/143 (6.9)	11/143 (7.6)	1.00
Hispanic	10/286 (3.5)	5/143 (3.5)	5/143 (3.5)	1.00
other	7/286 (2.4)	3/143 (2.1)	4/143 (2.8)	1.00
Diabetes mellitus, n (%)	45/286 (15.7)	18/143 (12.5)	27/143 (18.8)	0.14
Smoker, n (%)	112/286 (391)	54/143 (37.7)	58/143 (40.5)	0.62
Family history of moyamoya, n (%)	7/286 (2.4)	3/143 (2.1)	4/143 (2.8)	1.00
Underlying disease, n (%				
Sickle cell disease	14/286 (4.9)	8/148 (3.4)	6/143 (7.5)	0.78
Sickle cell trait	3/286 (1.0)	1/143 (0.7)	2/143 (1.4)	1.00
Neurofibromatosis	2/286 (0.7)	2/143 (1.4)	0/143 (0)	0.49
Procedure type, n (%)				
Indirect Revascularization	161/286 (56.2)	80/143 (55.9)	81/143 (56.6)	1.00
Direct Revascularization	147/286 (51.4)	71/143 (49.6)	76/143 (53.1)	0.55
Combined	22/286 (7.6)	8/143 (5.5)	14/143 (9.7)	0.26
Suzuki grade, n (%)				
I	12/286 (4.2)	6/143 (4.2)	6/143 (4.2)	1.00
II	28/286 (9.7)	16/143 (11.1)	12/143 (8.3)	0.55
III	90/286 (31.4)	42/143 (29.3)	48/143 (33.5)	0.44
IV	98/286 (34.2)	49/143 (34.2)	49/143 (34.2)	1.00
V	42/286 (14.6)	22/143 (15.3)	20/143 (13.9)	0.86
VI	17/286 (5.9)	8/143 (5.5)	9/143 (6.2)	1.00
Surgery side, n (%)				
Right hemisphere	153/286 (53.5)	67/143 (53.1)	66/143 (53.8)	0.90
Left hemisphere	133/286 (46.5)	67/143 (46.8)	66/143 (46.1)	0.90
Follow-up (months), median months (IQR)	16 (6-56)	21 (7-58)	15 (4-51)	0.27
mRS (0-2) on admission, n (%)	255/286 (89.1)	128/143 (89.5)	127/143 (88.8)	1.00
Incidental, n (%)	61/286 (21.3)	33/143 (23.0)	28/143 (19.5)	0.56
Stroke, n%				
Ischemic stroke	161286 (56.2)	79/143 (55.2)	82/143 (57.3)	0.72
TIA	77/286 (21.3)	38/143 (23.0)	39/143 (19.5)	1.00
Intraventricular hemorrhage	6/286 (2.1)	2/143 (1.4)	4/143 (2.8)	0.68
Intracerebral hemorrhage	10/286 (3.5)	6/143 (4.2)	4/143 (2.8)	0.74
Subarachnoid hemorrhage	20/286 (6.9)	10/143 (6.9)	10/143 (6.9)	1.00

**Table 4:** Comparison of outcomes between matched patients with and without hypertension

	With hypertension = 143	Without hypertension = 143	Effect variable	Value (95% CI)	*P*- value
Symptomatic stroke, n (%)	15/134 (11.1)	10/129 (7.7)	OR	1.5 (0.64 to 3.47)	0.34
Symptomatic ischemic stroke, n (%)	14/134 (10.4)	9/129 (6.9)	OR	1.55 (0.64 to 3.73)	0.32
Symptomatic hemorrhagic stroke, n (%)	1/134 (0.75)	1/129 (0.78)	OR	0.96 (0.05 to 15.55)	0.97
Intraoperative complication, n (%)	12/143 (8.3)	9/143 (6.2)	OR	1.36 (0.55 to 3.34)	0.49
Perioperative stroke, n (%)	9/143 (6.2)	3/143 (2.1)	OR	3.13 (0.83 to 11.82)	0.09
Perioperative minor symptomatic stroke, n (%)	6/143 (4.2)	1/143 (0.7)	OR	6.21 (0.73 to 52.33)	0.09
Perioperative major symptomatic stroke, n (%)	3/143 (2.1)	2/143 (1.4)	OR	1.51 (0.24 to 9.17)	0.65
Good functional outcome at discharge, n (%)	133/143 (93.0)	132/143 (92.3)	OR	1.10 (0.45 to 2.69)	0.82
NIHSS at discharge, median (IQR)	0 (0-0)	0 (0-0)	Beta	-0.004 (-0.05 to 0.04)	0.86
Length of hospital stay, median (IQR)	4 (2-4)	3 (2-5)	Beta	0.02 (-0.04 to 0.05)	0.91
Follow-up stroke, n (%)	9/143 (6.2)	8/143 (5.5)	OR	1.13 (0.42 to 3.02)	0.80
Follow-up ischemic stroke, n (%)	9/143 (6.2)	8/143 (5.5)	OR	1.13 (0.42 to 3.02)	0.80
Follow-up hemorrhagic stroke, n (%)	0/143 (0)	1/143 (0.7)	-	-	1.00

### Cox proportional hazard model

After adjusting the model to age, smoking, Suzuki grade, procedure type, diabetes mellitus, underlying disease, surgery side, and incidental MMD, there was no significant difference between the hypertension group and non-hypertension group in terms of symptomatic stroke (HR 1.33; 95% CI: 0.69-2.56, *p =* 0.38), and follow-up stroke (HR 0.90; 95% CI: 0.43-1.87, *p =* 0.78) (Supplementary Table 1).

## Discussion

In this study, hypertensive patients had a higher rate of symptomatic stroke, both ischemic and perioperative, compared to non-hypertensive patients. However, after propensity score matching, these differences did not reach statistical significance, indicating that confounding factors such as age, diabetes mellitus, and smoking status—more prevalent in the hypertensive group—may have contributed to the increased stroke risk observed in the unmatched analysis. Additionally, our adjusted cox proportional analysis showed no significant difference between the two groups in symptomatic and follow-up stroke rates.

Hypertension is a well-established major risk factor for numerous cardiovascular disorders, including stroke, due to its profound adverse effects on cerebral vascular structure and function [22]. Previous studies have emphasized that hypertension accelerates atherogenesis and is associated with increased cardiovascular morbidity [3, 7, 8, 18].

A meta-analysis was done by Wei et al. [25] to investigate the risk factors for postoperative stroke in MMD patients. Hypertension was found not to be associated with increased risk of postoperative stroke. Our study aligns with these findings, where hypertension was found not to be associated with symptomatic stroke, perioperative stroke, follow-up stroke or functional outcomes.

In a study by Ma et al. which investigated the effect of hypertension on moyamoya patients, they reported a higher rate of unfavorable outcomes and postoperative complications in hypertensive moyamoya patients [16]. In contrast, our study showed no significant differences between hypertensive moyamoya patients and non-hypertensive moyamoya patients in postoperative complications or functional outcomes.

Another study by Wang et al. which analyzed the associations between clinical risk factors and long-term outcomes in moyamoya patients found that hypertension was positively associated with follow-up stroke [24]. However, our study still showed no significant differences between hypertensive moyamoya patients and non-hypertensive moyamoya patients regarding follow-up stroke (whether ischemic or hemorrhagic), both before and after PSM.

The differences between our study and those proposed by Ma et al. [16] and Wang et al. [24] can be explained by the fact that their studies were conducted exclusively on Chinese populations. Although Chinese populations may have lower rates of hypertension compared to the American population, this lower prevalence can lead to lower awareness and control, resulting in higher complication rates [9, 10, 28]. Our study included populations from different ethnicities, with Caucasian populations being the most common. This suggests that treatment protocols and patient demographics can significantly impact outcomes in hypertensive Moyamoya patients.

This study has several limitations that should be considered. First, as a retrospective analysis, it is inherently subject to biases related to data collection and interpretation. Second, our focus was primarily on preoperative blood pressure values, without comprehensive monitoring of intraoperative and postoperative blood pressure variations, which could influence outcomes. Also, data on the severity of hypertension and the degree of its medical management were not uniformly available across all centers, limiting our ability to stratify these variables in our analysis. Additionally, the data were collected from multiple centers, leading to potential variability in clinical practices and patient management protocols. Third, the median follow-up period of 17 months is relatively short, which may limit the ability to capture long-term outcomes. Lastly, while propensity score matching was employed to balance baseline characteristics which provides a more accurate comparison between the groups, unmeasured confounders and reduction in statistical power may still affect the results.

## Conclusion

In conclusion, hypertensive, and non-hypertensive patients with MMD showed no significant differences in symptomatic stroke rates, perioperative strokes, or functional outcomes. Proper management can lead to comparable recovery in both groups. Further research is needed to optimize treatment strategies for hypertensive Moyamoya patients.

## Supplementary Information

Below is the link to the electronic supplementary material.

Supplementary Material 1

## Data Availability

Data can be provided on reasonable request from authors.
