# Supplementary material for: The impact of hypertension on clinical outcomes in moyamoya disease: a multicenter, propensity score-matched analysis
Source: Acta Neurochir (Wien). 2024 Sep 13;166(1):366. doi: 10.1007/s00701-024-06254-0 (PMC11399283; doi:10.1007/s00701-024-06254-0)
Supplement: Supplementary file 1 — Supplementary Material 1 [file 701_2024_6254_MOESM1_ESM.docx]

|  | **Cox proportional hazard**  **(Unadjusted Model)** | | | **Cox proportional hazard**  **(Adjusted Model*)** | | |
| --- | --- | --- | --- | --- | --- | --- |
| **Outcome** | HR | CI (95%) | P-value | HR | CI (95%) | P-value |
| **Symptomatic stroke** | 2.77 | (1.55 to 4.97) | 0.001 | 1.33 | (0.69 to 2.56) | 0.38 |
| **Follow-up stroke** | 1.58 | (0.83 to 3.02) | 0.16 | 0.90 | (0.43 to 1.87) | 0.78 |
| *All estimate were adjusted for age, smoking, Suzuki grade, procedure type, diabetes mellitus, underlying disease, surgery side, and incidental MMD. | | | | | | |

**Supplementary Table 1: Cox Proportional Hazard Model**
